# Supplementary material for: Complete NMR assignment for 275 of the most common dipeptides in intrinsically disordered proteins
Source: Sci Data. 2026 Jun 11;13:1348. doi: 10.1038/s41597-026-07602-8 (PMC13612823; doi:10.1038/s41597-026-07602-8)
Supplement: Supplementary file 1 [file 41597_2026_7602_MOESM1_ESM.pdf]

Supplementary information: Complete NMR assignment for 275 of the most common dipeptides in intrinsically disordered proteins

Tobias Rindfleisch[1,2,3,*], **Emilie Fjeldberg Taule**[2], **Markus S. Miettinen**[1,2,*,†], **and Jarl Underhaug**[2,*,†]

[1]Computational Biology Unit, Department of Informatics, University of Bergen, 5008 Bergen, Norway
[2]Department of Chemistry, University of Bergen, 5007 Bergen, Norway
[3]Max Planck Institute of Molecular Plant Physiology, 14476 Potsdam, Germany
[*]corresponding authors: Tobias Rindfleisch (tobias.rindfleisch@uib.no), Markus S. Miettinen (markus.miettinen@iki.fi), Jarl Underhaug (jarl.underhaug@uib.no)
[†]these authors contributed equally to this work

Data availability

The chemical shift datasets for 275 of the most common dipeptides in IDPs, along with the corresponding NMR spectra, are deposited in the Edmond Open Research Data Repository (edmond.mpg.de) at:

https://doi.org/10.17617/3.CBCNQG

Supplementary Figures

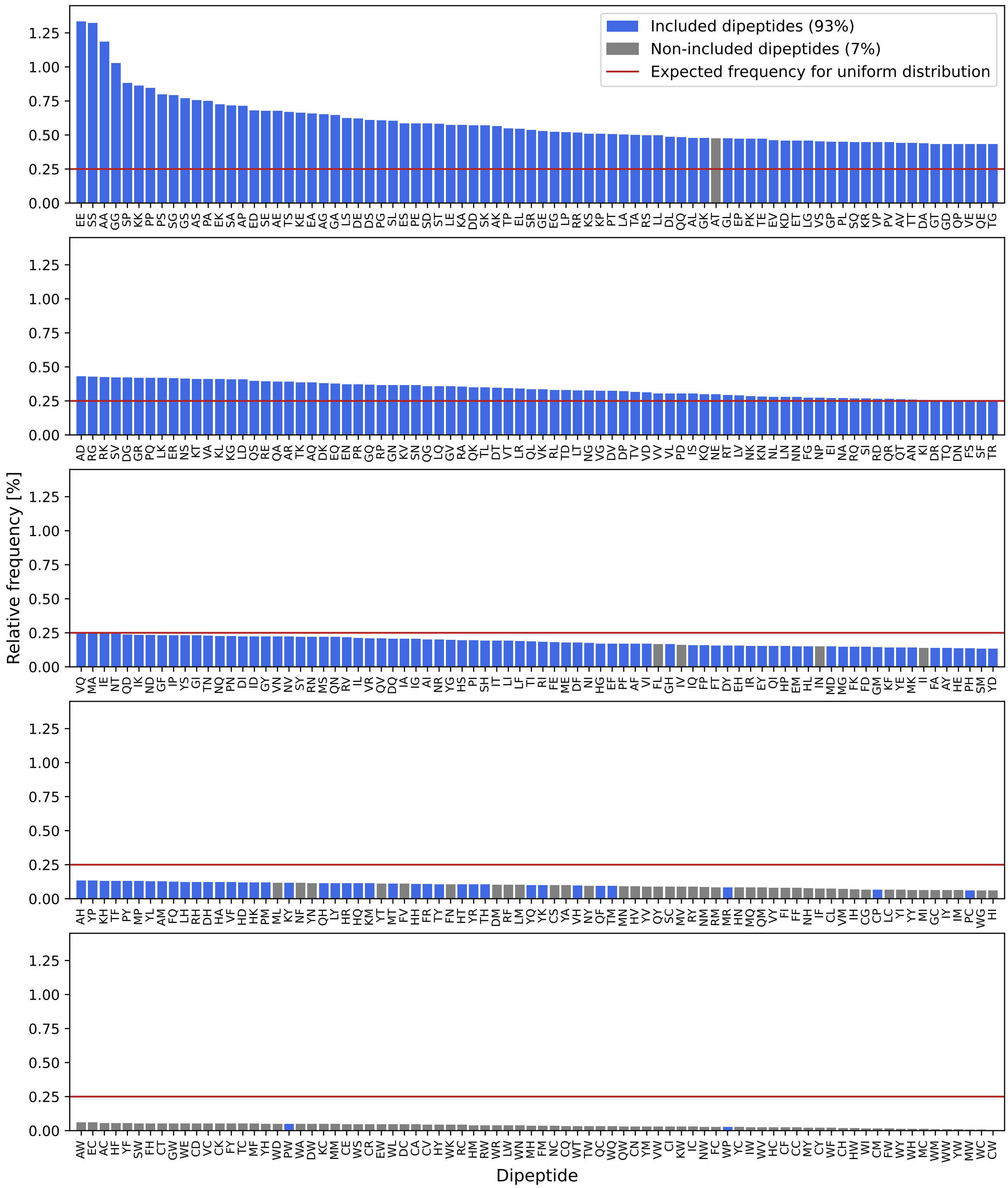

Figure S1. Relative frequencies of the 400 canonical dipeptides in the IDP proteome, as derived from the DisProt[1,2] database, compared to the uniform distribution (red line). Dipeptides included in the dataset are shown in blue and non-included ones in gray. The total frequency (%) is shown in the legend key for both populations. Additional details, including the relative and absolute frequencies, are provided in Table S2.

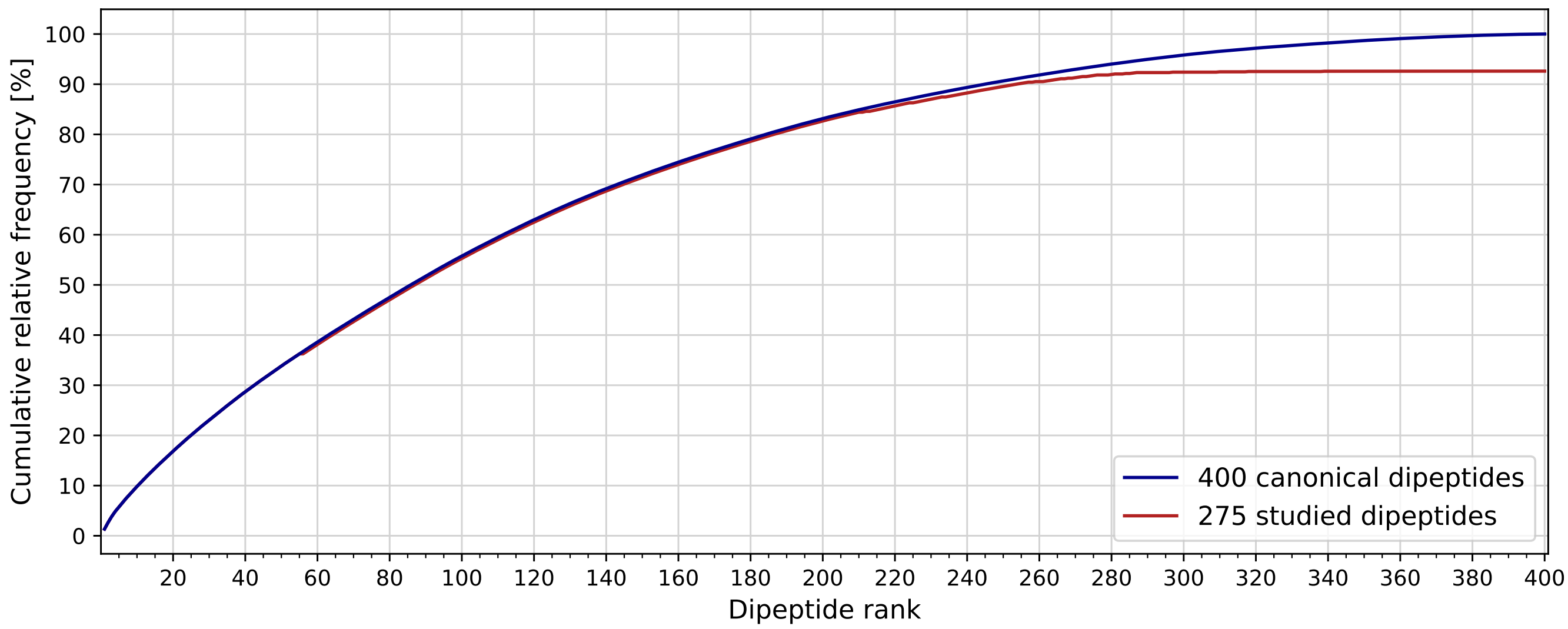

Figure S2. The cumulative distribution functions of the 275 studied (red), and all the 400 canonical dipeptides (blue) in the IDP proteome, derived from the DisProt[1,2] database, plotted as a function of the dipeptide rank (Figure 1 and Table S2).

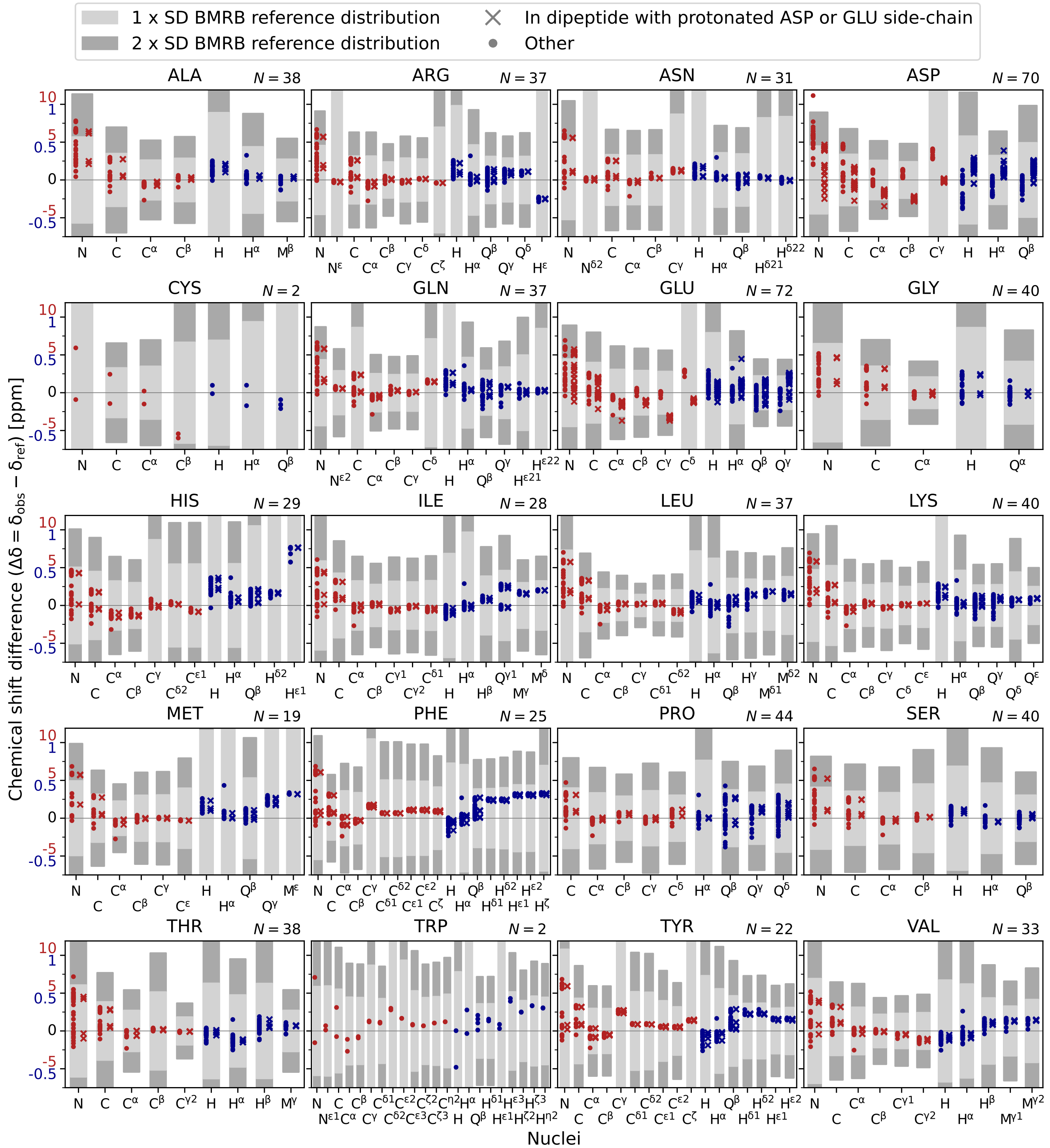

Figure S3. Residue-specific panels show the distributions of $\Delta\delta = \delta_{obs} - \delta_{ref}$ for each nucleus, defined as the difference between the observed chemical shifts (δ_{obs}) and the BMRB reference values[3] (δ_{ref}). Each panel has two *y*-axes, as the ^{1}H $\Delta\delta$ (blue) span a different range than the ^{13}C/^{15}N $\Delta\delta$ (red). Shaded regions indicate the BMRB reference standard deviation (SD) intervals[3]: $\pm$1SD (light grey) and $\pm$2SD (dark grey). *N* is the total number of observations per residue; dipeptides with protonated and deprotonated ASP or GLU side-chains are considered separately. ^{1}H resonances are classified as H, Q, and M: H represents CH protons with one data point; Q corresponds to CH_2 groups, for which the two protons are shown as separate data points; and M denotes methyl protons, generally yielding a single averaged chemical shift. Cross-symbols represent residues included in dipeptides with a protonated ASP or GLU side-chain, dots show residues in dipeptides with protonation states corresponding to those expected at pH 7 (ASP and GLU side-chains deprotonated).

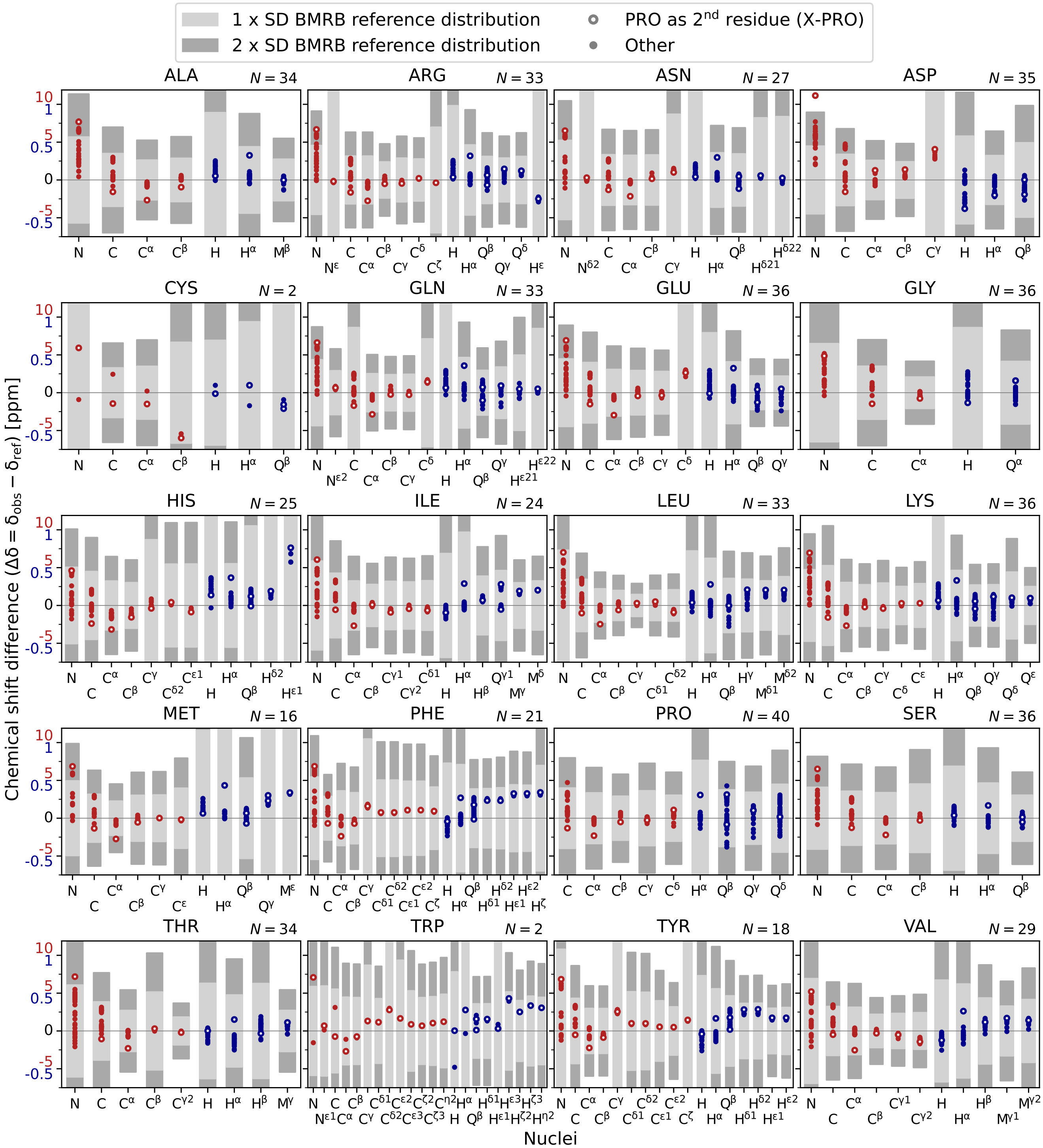

Figure S4. Dipeptides with protonation states corresponding to those expected at pH 7: Residue-specific panels show the distributions of $\Delta\delta = \delta_{obs} - \delta_{ref}$ for each nucleus, defined as the difference between the observed chemical shifts (δ_{obs}) and the BMRB reference values[3] (δ_{ref}). Each panel has two *y*-axes, as the ^{1}H $\Delta\delta$ (blue) span a different range than the ^{13}C/^{15}N $\Delta\delta$ (red). Shaded regions indicate the BMRB reference standard deviation (SD) intervals[3]: ±1SD (light grey) and ±2SD (dark grey). *N* is the total number of observations per residue. ^{1}H resonances are classified as H, Q, and M: H represents CH protons with one data point; Q corresponds to CH_2 groups, for which the two protons are shown as separate data points; and M denotes methyl protons, generally yielding a single averaged chemical shift. Circles indicate residues followed by proline (X–PRO motif) and dots represent all others.

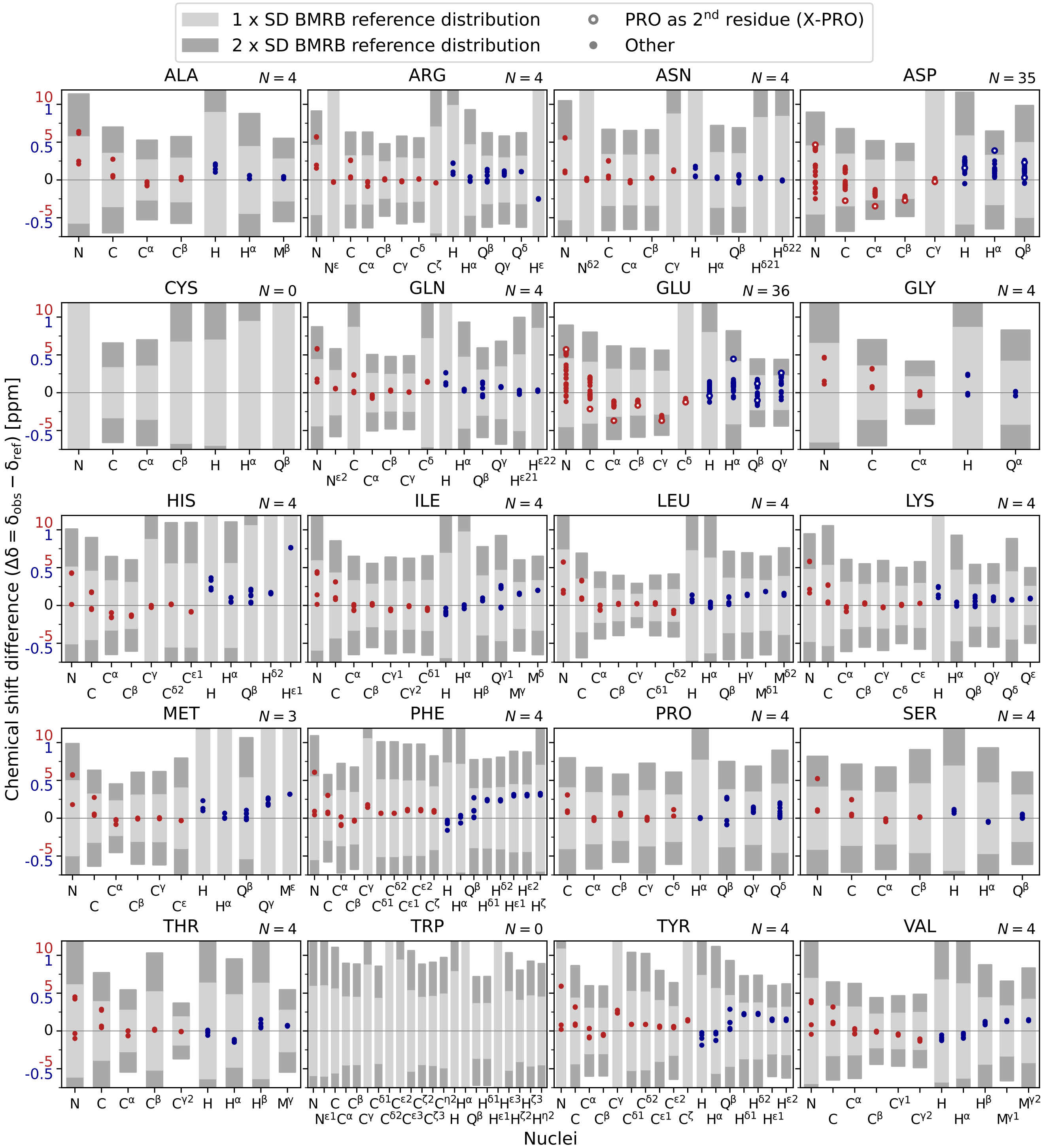

Figure S5. ASP or GLU containing dipeptides with protonated side-chains: Residue-specific panels show the distributions of $\Delta\delta = \delta_{obs} - \delta_{ref}$ for each nucleus, defined as the difference between the observed chemical shifts (δ_{obs}) and the BMRB reference values[3] (δ_{ref}). Each panel has two y-axes, as the ^{1}H $\Delta\delta$ (blue) span a different range than the ^{13}C/^{15}N $\Delta\delta$ (red). Shaded regions indicate the BMRB reference standard deviation (SD) intervals[3]: ±1SD (light grey) and ±2SD (dark grey). N is the total number of observations per residue. ^{1}H resonances are classified as H, Q, and M: H represents CH protons with one data point; Q corresponds to CH_2 groups, for which the two protons are shown as separate data points; and M denotes methyl protons, generally yielding a single averaged chemical shift. Circles indicate residues followed by proline (X–PRO motif) and dots represent all others.

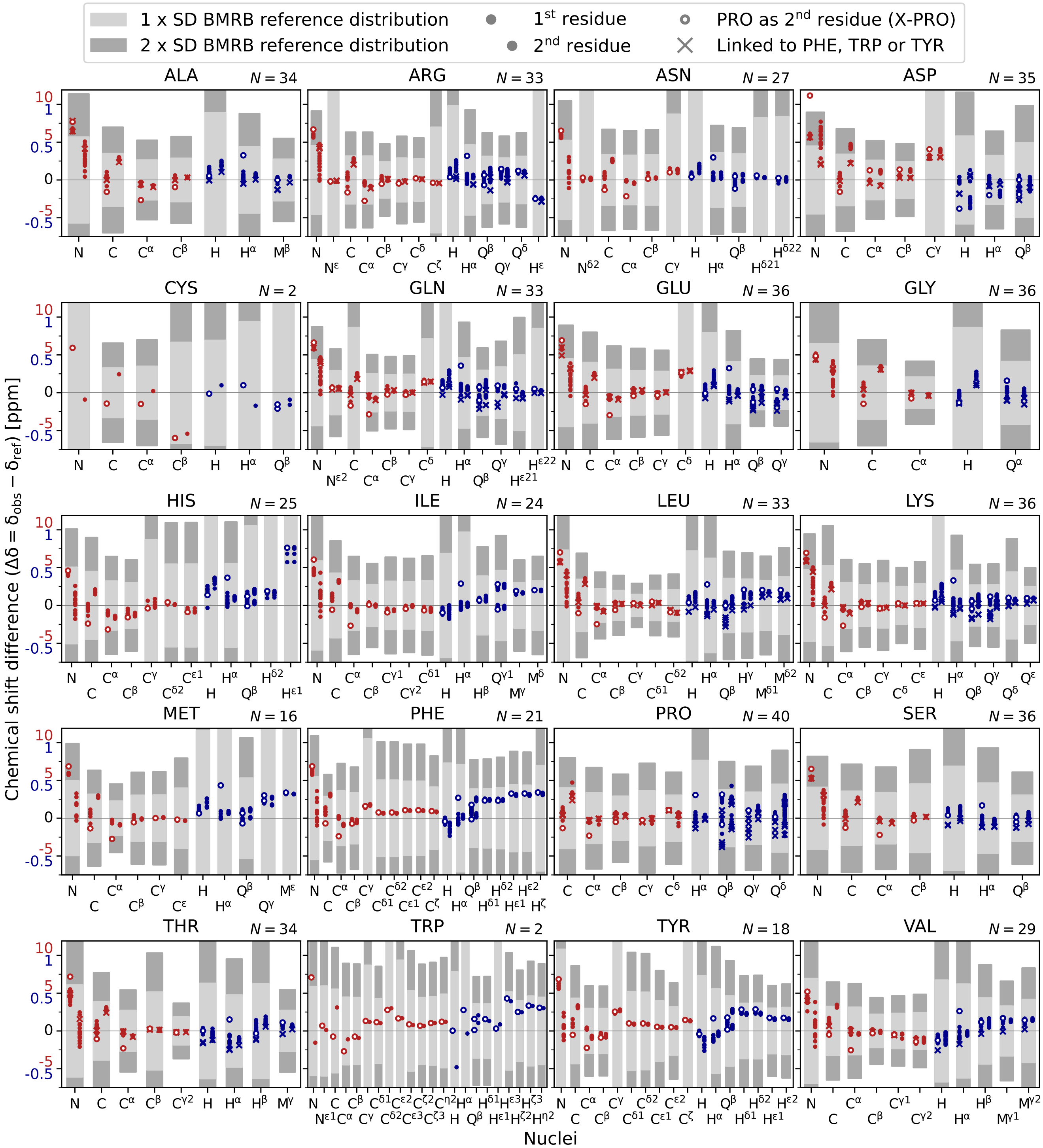

Figure S6. Dipeptides with protonation states corresponding to those expected at pH 7: Residue-specific panels display the distributions of $\Delta\delta = \delta_{obs} - \delta_{ref}$ for each nucleus, defined as the difference between the observed chemical shifts (δ_{obs}) and the BMRB reference values[3] (δ_{ref}). Each panel includes two y-axes, as the ^{1}H $\Delta\delta$ (blue) span a narrower range than the $^{13}C/^{15}N$ $\Delta\delta$ (red). Shaded regions mark the BMRB reference standard deviation (SD) intervals[3]: ±1SD (light grey) and ±2SD (dark grey). N is the total number of observations per residue. ^{1}H resonances are classified as H, Q, and M: H represents CH protons with one data point; Q corresponds to CH_2 groups, for which the two protons are shown as separate data points; and M denotes methyl protons, generally yielding a single averaged chemical shift. Data points corresponding to residues in the first position of the dipeptide are plotted on the left, while those in the second position are plotted on the right. Circles indicate residues followed by proline (X–PRO motif), dots represent all others, and cross-symbols mark residues linked to aromatic residues (PHE, TRP, or TYR).

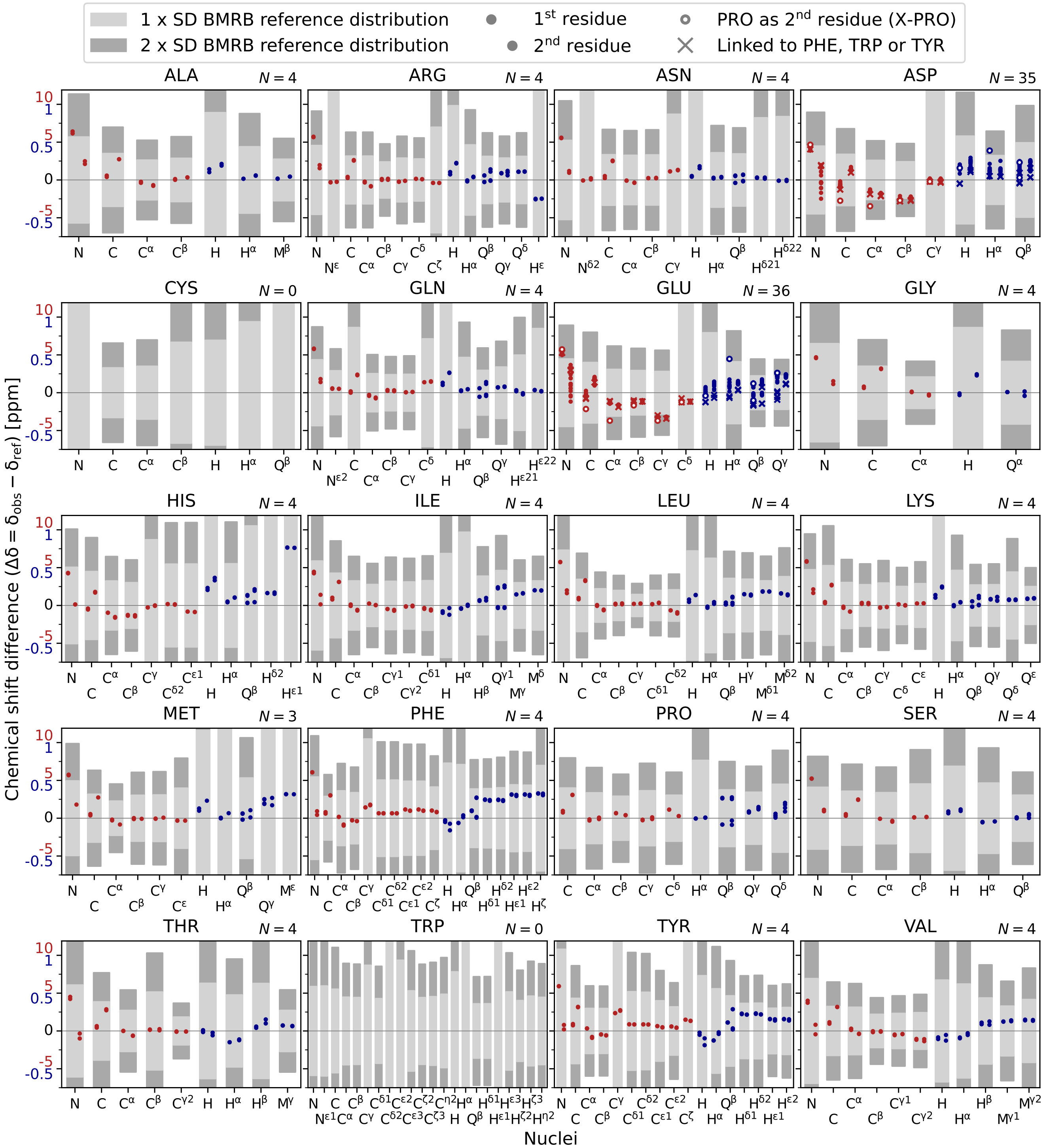

Figure S7. ASP or GLU containing dipeptides with protonated side-chains: Residue-specific panels display the distributions of $\Delta\delta = \delta_{obs} - \delta_{ref}$ for each nucleus, defined as the difference between the observed chemical shifts (δ_{obs}) and the BMRB reference values[3] (δ_{ref}). Each panel includes two *y*-axes, as the ^{1}H $\Delta\delta$ (blue) span a narrower range than the ^{13}C/^{15}N $\Delta\delta$ (red). Shaded regions mark the BMRB reference standard deviation (SD) intervals[3]: ±1SD (light grey) and ±2SD (dark grey). *N* is the total number of observations per residue. ^{1}H resonances are classified as H, Q, and M: H represents CH protons with one data point; Q corresponds to CH_2 groups, for which the two protons are shown as separate data points; and M denotes methyl protons, generally yielding a single averaged chemical shift. Data points corresponding to residues in the first position of the dipeptide are plotted on the left, while those in the second position are plotted on the right. Circles indicate residues followed by proline (X–PRO motif), dots represent all others, and cross-symbols mark residues linked to aromatic residues (PHE, TRP, or TYR).

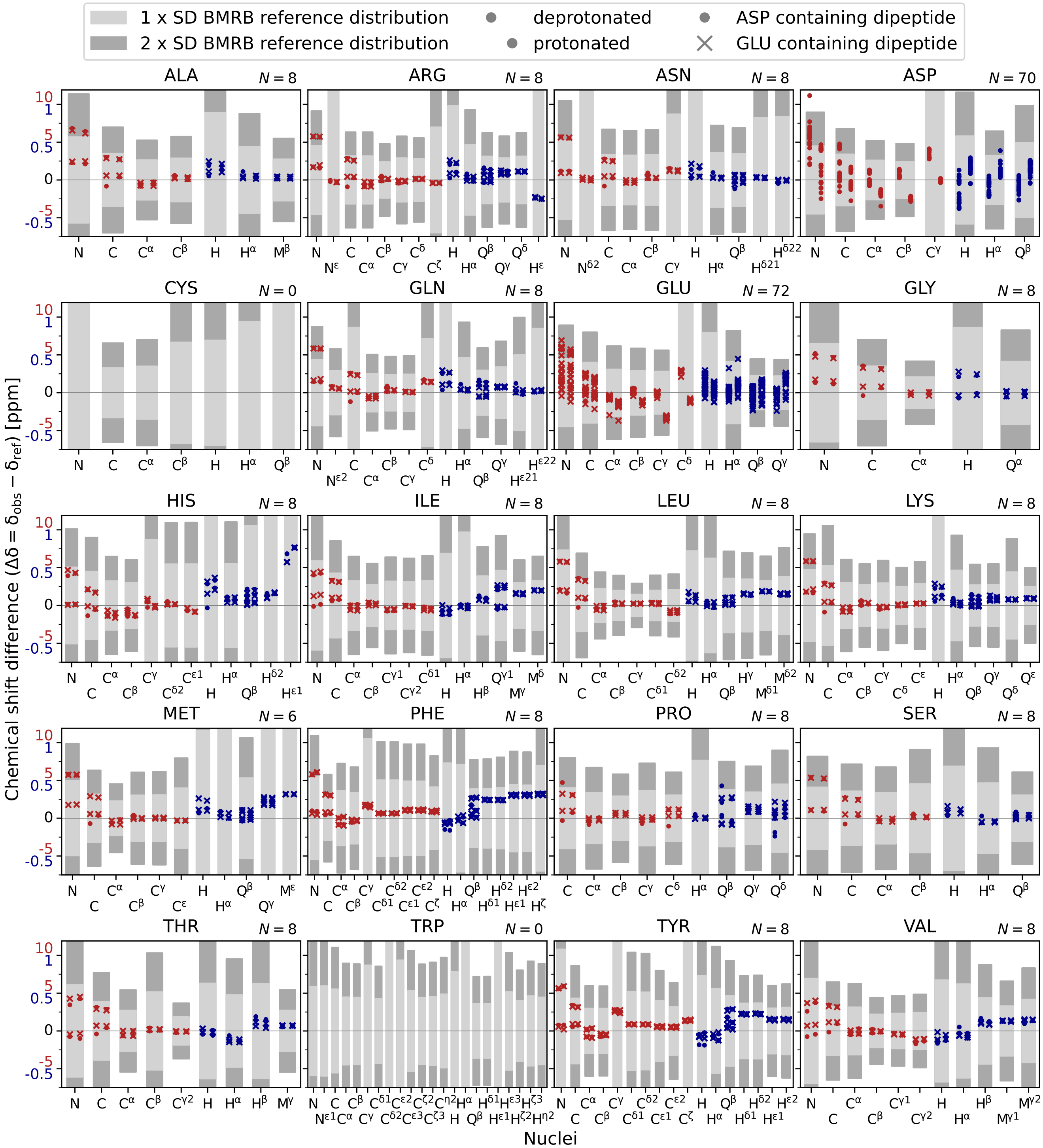

Figure S8. Protonation effects in ASP and GLU containing dipeptides: Residue-specific panels display the distributions of $\Delta\delta = \delta_{obs} - \delta_{ref}$ for each nucleus, defined as the difference between the observed chemical shifts (δ_{obs}) and the BMRB reference values[3] (δ_{ref}). Each panel includes two y-axes, as the ^{1}H $\Delta\delta$ (blue) span a narrower range than the ^{13}C/^{15}N $\Delta\delta$ (red). Shaded regions mark the BMRB reference standard deviation (SD) intervals[3]: $\pm$1SD (light grey) and $\pm$2SD (dark grey). N is the total number of observations per residue. ^{1}H resonances are classified as H, Q, and M: H represents CH protons with one data point; Q corresponds to CH_2 groups, for which the two protons are shown as separate data points; and M denotes methyl protons, generally yielding a single averaged chemical shift. Data points corresponding to dipeptides with deprotonated GLU/ASP are plotted on the left, while those with protonated GLU/ASP are plotted on the right. Circles indicate ASP-containing dipeptides and cross-symbols mark dipeptides including GLU.

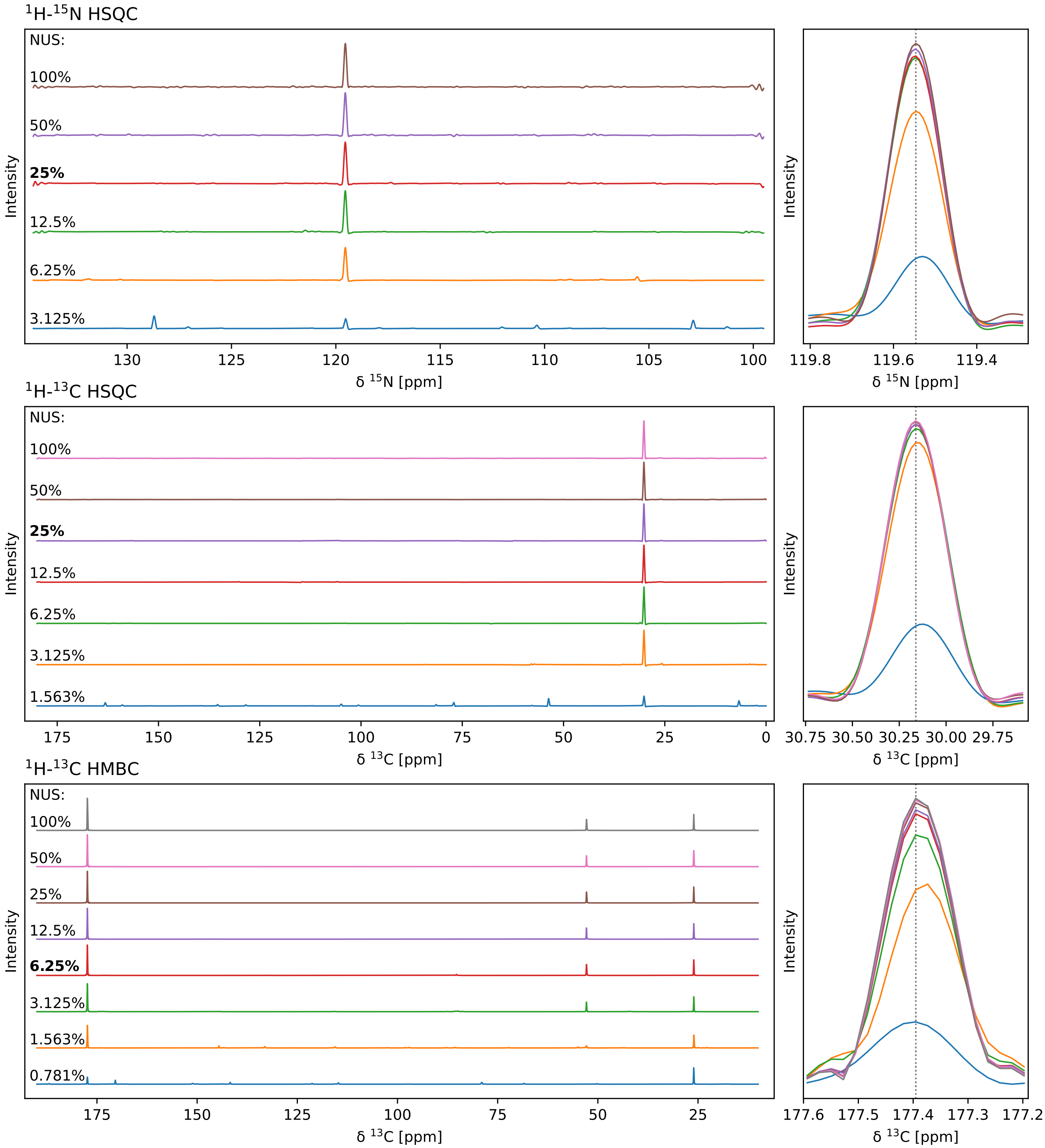

Figure S9. Assessment of NUS-related effects, exemplarily demonstrated for the dipeptide AE. To evaluate potential artifact occurrence (left panels) and perturbations of chemical shift (δ) positions (right panels), we show 1D columns extracted from the corresponding 2D spectra at ^{1}H = 8.38 ppm for the ^{1}H–^{15}N HSQC, and at ^{1}H = 2.48 ppm for the ^{1}H–^{13}C HSQC and ^{1}H–^{13}C HMBC, using NUS fractions from 0.781% to 100%. The results show no effect on chemical-shift positions for the NUS fractions used in this study (highlighted in bold). Furthermore, peak intensities at 100% sampling (uniform sampling) and at the applied NUS fractions differ only marginally, and no spectral artifacts are observed, except for a single marginal artificial peak in the HMBC dataset.

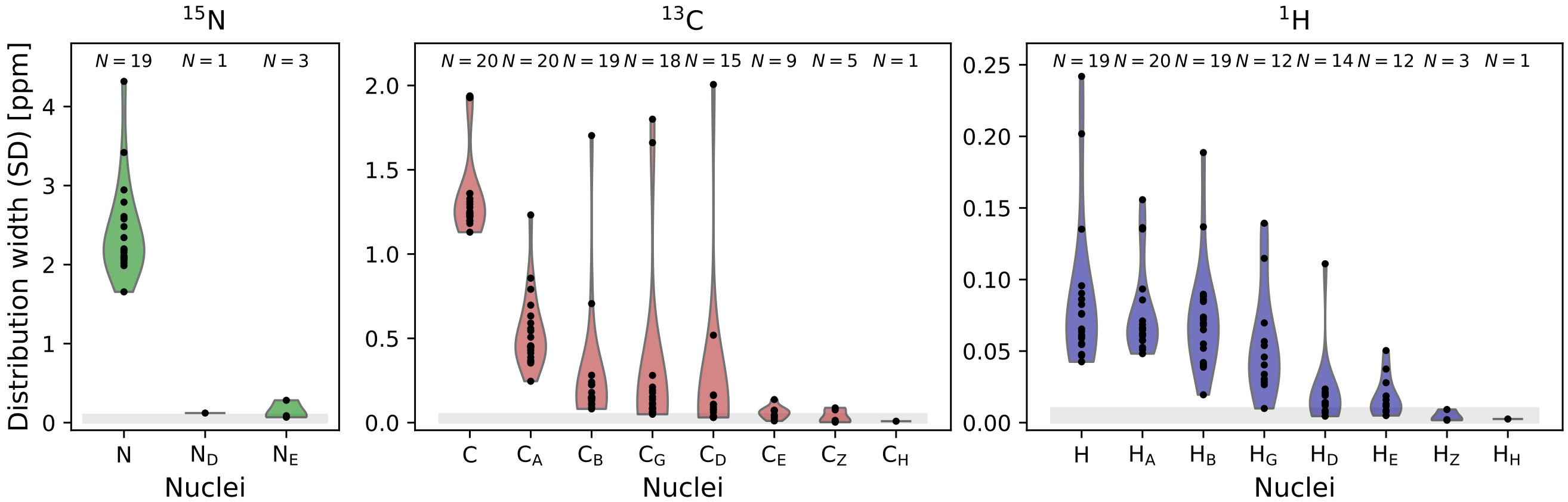

Figure S10. For every residue–atom-type combination plotted in Figure 2 (main text), the standard deviation ($SD_{res,at}$, with res: residue and at: atom type) of the corresponding data points (Figure 2) was calculated and is shown as a black dot. The resulting $SD_{res,at}$ values were then grouped according to the atom type (x-axis) and are plotted separately for ^{15}N, ^{13}C, and ^{1}H. For every atom type, the underlying distributions of computed $SD_{res,at}$-values are visualized as violins, with N indicating the number of individual observations per distribution. Broader distributions or higher $SD_{res,at}$ values reflect greater chemical shift variability for the corresponding atom types. Gray-shaded regions indicate the typical assignment uncertainties of the respective nuclei (^{15}N: 0.1 ppm, ^{13}C: 0.05 ppm, and ^{1}H: 0.01 ppm).

Supplementary Tables

Table S1. Overview of NMR instruments used for individual dipeptide measurements.

Instrument	Analyzed dipeptides
Ascend 600 MHz with QCI-P CryoProbe	AA, AD, AE, AG, AK, AL, AN, AP, AQ, AR, AS, AV, CP, DA, DD, DE, DG, DH, DI, DK, DL, DN, DP, DQ, DR, DS, DT, DV, EA, ED, EE, EF, EG, EH, EI, EK, EL, EN, EP, EQ, ER, ES, ET, EV, EY, FA, FG, FK, FP, FS, GA, GD, GE, GF, GG, GH, GI, GK, GL, GN, GP, GQ, GR, GS, GT, GV, GY, HE, HG, HH, HK, HL, HP, HQ, IA, ID, IE, IG, IK, IL, IP, IS, KA, KD, KE, KF, KG, KH, KI, KK, KL, KN, KP, KQ, KR, KS, KT, KV, LA, LD, LE, LF, LG, LI, LK, LL, LN, LP, LQ, LR, LS, LT, LV, MA, MD, ME, MG, MP, NA, ND, NE, NG, NK, NL, NP, NS, NT, NV, PA, PC, PD, PE, PF, PG, PH, PI, PK, PL, PN, PP, PQ, PR, PS, PT, PW, PY, QA, QD, QE, QG, QK, QL, QN, QP, QR, QS, QT, QV, RA, RD, RE, RG, RK, RL, RP, RQ, RS, RT, SA, SD, SE, SF, SG, SH, SI, SK, SL, SN, SP, SQ, SR, SS, ST, SV, SY, TA, TD, TE, TG, TH, TK, TL, TM, TN, TP, TQ, TR, TS, TT, TV, VA, VD, VE, VF, VG, VH, VK, VL, VP, VQ, VR, VT, VV, YE, YG, YP
Ascend 850 MHz with TCI CryoProbe	AF, AH, AI, AM, AY, DF, DY, EM, FD, FE, FQ, FR, FT, GM, HA, HD, HR, HS, HT, IQ, IR, IT, KM, KY, LH, LY, MK, MR, MS, MT, NN, NQ, NR, PM, PV, QF, QH, QI, RH, RI, RN, RR, RV, SM, TF, TI, TY, VI, VN, WP, YD, YK, YL, YQ, YR, YS

Table S2. The relative frequencies (rel. freq.) of all the 400 canonical dipeptides in the DisProt[1,2] database (that contains experimentally-confirmed IDP sequences); their absolute occurrence (abs. occurr.) in DisProt; and their relative frequency in the dataset presented here (dipeptides not included are indicated as "–"). Entries are sorted by the relative frequency / rank (left; according to Figure 1) and by the dipeptide in alphabetical order (right).

Sorted by relative frequency (rank)					Sorted alphabetically by dipeptide name				
Rank	Dipep-tide	Rel. freq. in DisProt [%]	Abs. occurr. in DisProt	Rel. freq. in dataset [%]	Rank	Dipep-tide	Rel. freq. in DisProt [%]	Abs. occurr. in DisProt	Rel. freq. in dataset [%]
1	EE	1.3333	2955	1.4397	3	AA	1.1866	2630	1.2813
2	SS	1.3224	2931	1.4280	323	AC	0.0559	124	–
3	AA	1.1866	2630	1.2813	81	AD	0.4309	955	0.4653
4	GG	1.0296	2282	1.1118	18	AE	0.6768	1500	0.7308
5	SP	0.8816	1954	0.9520	209	AF	0.1696	376	0.1832
6	KK	0.8636	1914	0.9325	22	AG	0.6520	1445	0.7040
7	PP	0.8464	1876	0.9140	241	AH	0.1331	295	0.1437
8	PS	0.7995	1772	0.8633	191	AI	0.2008	445	0.2168
9	SG	0.7927	1757	0.8560	37	AK	0.5653	1253	0.6105
10	GS	0.7706	1708	0.8321	54	AL	0.4778	1059	0.5159
11	AS	0.7553	1674	0.8156	248	AM	0.1263	280	0.1364
12	PA	0.7508	1664	0.8107	153	AN	0.2585	573	0.2792
13	EK	0.7269	1611	0.7849	15	AP	0.7129	1580	0.7698
14	SA	0.7178	1591	0.7751	101	AQ	0.3853	854	0.4161
15	AP	0.7129	1580	0.7698	99	AR	0.3907	866	0.4219
16	ED	0.6808	1509	0.7352	11	AS	0.7553	1674	0.8156
17	SE	0.6786	1504	0.7328	56	AT	0.4765	1056	–
18	AE	0.6768	1500	0.7308	72	AV	0.4422	980	0.4775
19	TS	0.6687	1482	0.7220	321	AW	0.0591	131	–
20	KE	0.6632	1470	0.7162	236	AY	0.1376	305	0.1486
21	EA	0.6578	1458	0.7103	350	CA	0.0456	101	–
22	AG	0.6520	1445	0.7040	383	CC	0.0230	51	–
23	GA	0.6461	1432	0.6977	331	CD	0.0514	114	–
24	LS	0.6235	1382	0.6733	344	CE	0.0474	105	–

Continued on next page

Table S2 (continued)

Sorted by relative frequency (rank)					Sorted alphabetically by dipeptide name				
Rank	Dipep-tide	Rel. freq. in DisProt [%]	Abs. occurr. in DisProt	Rel. freq. in dataset [%]	Rank	Dipep-tide	Rel. freq. in DisProt [%]	Abs. occurr. in DisProt	Rel. freq. in dataset [%]
25	DE	0.6208	1376	0.6704	382	CF	0.0235	52	–
26	DS	0.6091	1350	0.6577	309	CG	0.0663	147	–
27	PG	0.6087	1349	0.6572	387	CH	0.0180	40	–
28	SL	0.6055	1342	0.6538	372	CI	0.0298	66	–
29	ES	0.5865	1300	0.6334	333	CK	0.0510	113	–
30	PE	0.5865	1300	0.6334	306	CL	0.0735	163	–
31	SD	0.5843	1295	0.6309	390	CM	0.0162	36	–
32	ST	0.5811	1288	0.6275	369	CN	0.0307	68	–
33	LE	0.5753	1275	0.6212	310	CP	0.0659	146	0.0711
34	KA	0.5730	1270	0.6187	363	CQ	0.0334	74	–
35	DD	0.5712	1266	0.6168	346	CR	0.0465	103	–
36	SK	0.5708	1265	0.6163	282	CS	0.0993	220	–
37	AK	0.5653	1253	0.6105	328	CT	0.0523	116	–
38	TP	0.5473	1213	0.5910	351	CV	0.0442	98	–
39	EL	0.5455	1209	0.5890	400	CW	0.0054	12	–
40	SR	0.5369	1190	0.5798	385	CY	0.0217	48	–
41	GE	0.5301	1175	0.5725	74	DA	0.4404	976	0.4755
42	EG	0.5247	1163	0.5666	349	DC	0.0456	101	–
43	LP	0.5211	1155	0.5627	35	DD	0.5712	1266	0.6168
44	RR	0.5175	1147	0.5588	25	DE	0.6208	1376	0.6704
45	KS	0.5098	1130	0.5505	204	DF	0.1778	394	0.1920
46	KP	0.5089	1128	0.5496	85	DG	0.4214	934	0.4550
47	PT	0.5076	1125	0.5481	252	DH	0.1223	271	0.1320
48	LA	0.5035	1116	0.5437	175	DI	0.2229	494	0.2407
49	TA	0.5004	1109	0.5403	102	DK	0.3804	843	0.4107
50	RS	0.4986	1105	0.5384	52	DL	0.4859	1077	0.5247
51	LL	0.4986	1105	0.5384	277	DM	0.1024	227	–
52	DL	0.4859	1077	0.5247	157	DN	0.2527	560	0.2728
53	QQ	0.4846	1074	0.5233	128	DP	0.3203	710	0.3459
54	AL	0.4778	1059	0.5159	188	DQ	0.2071	459	0.2236
55	GK	0.4774	1058	0.5155	155	DR	0.2545	564	0.2748
56	AT	0.4765	1056	–	26	DS	0.6091	1350	0.6577
57	GL	0.4746	1052	0.5125	117	DT	0.3479	771	0.3756
58	EP	0.4742	1051	0.5121	127	DV	0.3244	719	0.3503
59	PK	0.4724	1047	0.5101	341	DW	0.0487	108	–
60	TE	0.4715	1045	0.5091	217	DY	0.1552	344	0.1676
61	EV	0.4620	1024	0.4989	21	EA	0.6578	1458	0.7103
62	KD	0.4598	1019	0.4965	322	EC	0.0591	131	–
63	ET	0.4580	1015	0.4945	16	ED	0.6808	1509	0.7352
64	LG	0.4575	1014	0.4940	1	EE	1.3333	2955	1.4397
65	VS	0.4521	1002	0.4882	207	EF	0.1701	377	0.1837
66	GP	0.4507	999	0.4867	42	EG	0.5247	1163	0.5666
67	PL	0.4503	998	0.4862	218	EH	0.1552	344	0.1676
68	SQ	0.4489	995	0.4848	146	EI	0.2721	603	0.2938
69	KR	0.4471	991	0.4828	13	EK	0.7269	1611	0.7849
70	VP	0.4467	990	0.4823	39	EL	0.5455	1209	0.5890
71	PV	0.4467	990	0.4823	223	EM	0.1502	333	0.1622
72	AV	0.4422	980	0.4775	104	EN	0.3722	825	0.4019
73	TT	0.4408	977	0.4760	58	EP	0.4742	1051	0.5121

Continued on next page

Table S2 (continued)

Sorted by relative frequency (rank)					Sorted alphabetically by dipeptide name				
Rank	Dipep-tide	Rel. freq. in DisProt [%]	Abs. occurr. in DisProt	Rel. freq. in dataset [%]	Rank	Dipep-tide	Rel. freq. in DisProt [%]	Abs. occurr. in DisProt	Rel. freq. in dataset [%]
74	DA	0.4404	976	0.4755	103	EQ	0.3781	838	0.4083
75	GT	0.4345	963	0.4692	89	ER	0.4160	922	0.4492
76	GD	0.4345	963	0.4692	29	ES	0.5865	1300	0.6334
77	QP	0.4345	963	0.4692	63	ET	0.4580	1015	0.4945
78	VE	0.4340	962	0.4687	61	EV	0.4620	1024	0.4989
79	QE	0.4340	962	0.4687	347	EW	0.0465	103	–
80	TG	0.4327	959	0.4672	220	EY	0.1539	341	0.1661
81	AD	0.4309	955	0.4653	235	FA	0.1394	309	0.1505
82	RG	0.4277	948	0.4619	376	FC	0.0266	59	–
83	RK	0.4264	945	0.4604	229	FD	0.1462	324	0.1579
84	SV	0.4232	938	0.4570	202	FE	0.1814	402	0.1959
85	DG	0.4214	934	0.4550	303	FF	0.0790	175	–
86	GR	0.4210	933	0.4546	144	FG	0.2743	608	0.2962
87	PQ	0.4210	933	0.4546	327	FH	0.0528	117	–
88	LK	0.4196	930	0.4531	302	FI	0.0794	176	–
89	ER	0.4160	922	0.4492	228	FK	0.1462	324	0.1579
90	NS	0.4133	916	0.4463	211	FL	0.1678	372	–
91	KT	0.4115	912	0.4443	361	FM	0.0356	79	–
92	VA	0.4115	912	0.4443	273	FN	0.1056	234	–
93	KL	0.4115	912	0.4443	215	FP	0.1588	352	0.1715
94	KG	0.4088	906	0.4414	249	FQ	0.1236	274	0.1335
95	LD	0.4083	905	0.4409	271	FR	0.1087	241	0.1174
96	QS	0.3961	878	0.4278	158	FS	0.2495	553	0.2694
97	RE	0.3948	875	0.4263	216	FT	0.1557	345	0.1681
98	QA	0.3912	867	0.4224	269	FV	0.1101	244	–
99	AR	0.3907	866	0.4219	391	FW	0.0149	33	–
100	TK	0.3853	854	0.4161	334	FY	0.0510	113	–
101	AQ	0.3853	854	0.4161	23	GA	0.6461	1432	0.6977
102	DK	0.3804	843	0.4107	315	GC	0.0627	139	–
103	EQ	0.3781	838	0.4083	76	GD	0.4345	963	0.4692
104	EN	0.3722	825	0.4019	41	GE	0.5301	1175	0.5725
105	PR	0.3713	823	0.4010	168	GF	0.2328	516	0.2514
106	GQ	0.3691	818	0.3985	4	GG	1.0296	2282	1.1118
107	RP	0.3673	814	0.3966	212	GH	0.1674	371	0.1808
108	GN	0.3673	814	0.3966	171	GI	0.2306	511	0.2490
109	KV	0.3659	811	0.3951	55	GK	0.4774	1058	0.5155
110	SN	0.3659	811	0.3951	57	GL	0.4746	1052	0.5125
111	QG	0.3582	794	0.3868	230	GM	0.1457	323	0.1574
112	LQ	0.3578	793	0.3864	108	GN	0.3673	814	0.3966
113	GV	0.3564	790	0.3849	66	GP	0.4507	999	0.4867
114	RA	0.3546	786	0.3829	106	GQ	0.3691	818	0.3985
115	QK	0.3492	774	0.3771	86	GR	0.4210	933	0.4546
116	TL	0.3483	772	0.3761	10	GS	0.7706	1708	0.8321
117	DT	0.3479	771	0.3756	75	GT	0.4345	963	0.4692
118	VT	0.3438	762	0.3712	113	GV	0.3564	790	0.3849
119	LR	0.3415	757	0.3688	329	GW	0.0523	116	–
120	QL	0.3366	746	0.3635	177	GY	0.2224	493	0.2402
121	VK	0.3352	743	0.3620	253	HA	0.1223	271	0.1320
122	RL	0.3307	733	0.3571	381	HC	0.0244	54	–

Continued on next page

Table S2 (continued)

Sorted by relative frequency (rank)					Sorted alphabetically by dipeptide name				
Rank	Dipep-tide	Rel. freq. in DisProt [%]	Abs. occurr. in DisProt	Rel. freq. in dataset [%]	Rank	Dipep-tide	Rel. freq. in DisProt [%]	Abs. occurr. in DisProt	Rel. freq. in dataset [%]
123	TD	0.3298	731	0.3561	255	HD	0.1205	267	0.1301
124	LT	0.3276	726	0.3537	237	HE	0.1372	304	0.1481
125	NG	0.3276	726	0.3537	324	HF	0.0550	122	–
126	VG	0.3249	720	0.3508	206	HG	0.1705	378	0.1842
127	DV	0.3244	719	0.3503	270	HH	0.1092	242	0.1179
128	DP	0.3203	710	0.3459	320	HI	0.0596	132	–
129	TV	0.3154	699	0.3406	256	HK	0.1200	266	0.1296
130	VD	0.3136	695	0.3386	224	HL	0.1498	332	0.1618
131	VV	0.3050	676	0.3293	355	HM	0.0379	84	–
132	VL	0.3046	675	0.3289	298	HN	0.0835	185	–
133	PD	0.3041	674	0.3284	222	HP	0.1521	337	0.1642
134	IS	0.3032	672	0.3274	265	HQ	0.1132	251	0.1223
135	KQ	0.2982	661	0.3220	264	HR	0.1132	251	0.1223
136	NE	0.2978	660	0.3216	194	HS	0.1949	432	0.2105
137	RT	0.2924	648	0.3157	274	HT	0.1051	233	0.1135
138	LV	0.2901	643	0.3133	289	HV	0.0902	200	–
139	NK	0.2861	634	0.3089	388	HW	0.0171	38	–
140	KN	0.2815	624	0.3040	352	HY	0.0438	97	–
141	NL	0.2797	620	0.3021	189	IA	0.2066	458	0.2231
142	LN	0.2793	619	0.3016	374	IC	0.0289	64	–
143	NN	0.2784	617	0.3006	176	ID	0.2229	494	0.2407
144	FG	0.2743	608	0.2962	163	IE	0.2441	541	0.2636
145	NP	0.2743	608	0.2962	305	IF	0.0753	167	–
146	EI	0.2721	603	0.2938	190	IG	0.2057	456	0.2222
147	NA	0.2707	600	0.2923	308	IH	0.0690	153	–
148	RQ	0.2685	595	0.2899	234	II	0.1403	311	–
149	SI	0.2680	594	0.2894	166	IK	0.2351	521	0.2538
150	RD	0.2653	588	0.2865	185	IL	0.2130	472	0.2300
151	QR	0.2648	587	0.2860	317	IM	0.0618	137	–
152	QT	0.2617	580	0.2826	225	IN	0.1489	330	–
153	AN	0.2585	573	0.2792	169	IP	0.2319	514	0.2504
154	KI	0.2567	569	0.2772	214	IQ	0.1597	354	0.1725
155	DR	0.2545	564	0.2748	219	IR	0.1543	342	0.1666
156	TQ	0.2531	561	0.2733	134	IS	0.3032	672	0.3274
157	DN	0.2527	560	0.2728	197	IT	0.1918	425	0.2071
158	FS	0.2495	553	0.2694	213	IV	0.1611	357	–
159	SF	0.2486	551	0.2684	379	IW	0.0248	55	–
160	TR	0.2482	550	0.2680	316	IY	0.0623	138	–
161	VQ	0.2463	546	0.2660	34	KA	0.5730	1270	0.6187
162	MA	0.2445	542	0.2641	342	KC	0.0487	108	–
163	IE	0.2441	541	0.2636	62	KD	0.4598	1019	0.4965
164	NT	0.2432	539	0.2626	20	KE	0.6632	1470	0.7162
165	QD	0.2369	525	0.2558	231	KF	0.1417	314	0.1530
166	IK	0.2351	521	0.2538	94	KG	0.4088	906	0.4414
167	ND	0.2346	520	0.2533	243	KH	0.1317	292	0.1423
168	GF	0.2328	516	0.2514	154	KI	0.2567	569	0.2772
169	IP	0.2319	514	0.2504	6	KK	0.8636	1914	0.9325
170	YS	0.2310	512	0.2494	93	KL	0.4115	912	0.4443
171	GI	0.2306	511	0.2490	266	KM	0.1128	250	0.1218

Continued on next page

Table S2 (continued)

Sorted by relative frequency (rank)					Sorted alphabetically by dipeptide name				
Rank	Dipep-tide	Rel. freq. in DisProt [%]	Abs. occurr. in DisProt	Rel. freq. in dataset [%]	Rank	Dipep-tide	Rel. freq. in DisProt [%]	Abs. occurr. in DisProt	Rel. freq. in dataset [%]
172	TN	0.2288	507	0.2470	140	KN	0.2815	624	0.3040
173	NQ	0.2256	500	0.2436	46	KP	0.5089	1128	0.5496
174	PN	0.2251	499	0.2431	135	KQ	0.2982	661	0.3220
175	DI	0.2229	494	0.2407	69	KR	0.4471	991	0.4828
176	ID	0.2229	494	0.2407	45	KS	0.5098	1130	0.5505
177	GY	0.2224	493	0.2402	91	KT	0.4115	912	0.4443
178	VN	0.2224	493	0.2402	109	KV	0.3659	811	0.3951
179	NV	0.2220	492	0.2397	373	KW	0.0298	66	–
180	SY	0.2206	489	0.2382	259	KY	0.1169	259	0.1262
181	RN	0.2202	488	0.2378	48	LA	0.5035	1116	0.5437
182	MS	0.2197	487	0.2373	311	LC	0.0654	145	–
183	QN	0.2193	486	0.2368	95	LD	0.4083	905	0.4409
184	RV	0.2166	480	0.2339	33	LE	0.5753	1275	0.6212
185	IL	0.2130	472	0.2300	199	LF	0.1881	417	0.2032
186	VR	0.2089	463	0.2256	64	LG	0.4575	1014	0.4940
187	QV	0.2080	461	0.2246	250	LH	0.1232	273	0.1330
188	DQ	0.2071	459	0.2236	198	LI	0.1909	423	0.2061
189	IA	0.2066	458	0.2231	88	LK	0.4196	930	0.4531
190	IG	0.2057	456	0.2222	51	LL	0.4986	1105	0.5384
191	AI	0.2008	445	0.2168	279	LM	0.1011	224	–
192	NR	0.1999	443	0.2158	142	LN	0.2793	619	0.3016
193	YG	0.1990	441	0.2149	43	LP	0.5211	1155	0.5627
194	HS	0.1949	432	0.2105	112	LQ	0.3578	793	0.3864
195	PI	0.1940	430	0.2095	119	LR	0.3415	757	0.3688
196	SH	0.1936	429	0.2090	24	LS	0.6235	1382	0.6733
197	IT	0.1918	425	0.2071	124	LT	0.3276	726	0.3537
198	LI	0.1909	423	0.2061	138	LV	0.2901	643	0.3133
199	LF	0.1881	417	0.2032	358	LW	0.0365	81	–
200	TI	0.1854	411	0.2002	263	LY	0.1142	253	0.1233
201	RI	0.1850	410	0.1998	162	MA	0.2445	542	0.2641
202	FE	0.1814	402	0.1959	394	MC	0.0113	25	–
203	ME	0.1782	395	0.1924	226	MD	0.1489	330	0.1608
204	DF	0.1778	394	0.1920	203	ME	0.1782	395	0.1924
205	NI	0.1760	390	0.1900	336	MF	0.0505	112	–
206	HG	0.1705	378	0.1842	227	MG	0.1466	325	0.1583
207	EF	0.1701	377	0.1837	360	MH	0.0361	80	–
208	PF	0.1696	376	0.1832	314	MI	0.0641	142	–
209	AF	0.1696	376	0.1832	233	MK	0.1408	312	0.1520
210	VI	0.1687	374	0.1822	258	ML	0.1178	261	–
211	FL	0.1678	372	–	343	MM	0.0483	107	–
212	GH	0.1674	371	0.1808	288	MN	0.0925	205	–
213	IV	0.1611	357	–	246	MP	0.1299	288	0.1403
214	IQ	0.1597	354	0.1725	299	MQ	0.0830	184	–
215	FP	0.1588	352	0.1715	297	MR	0.0839	186	0.0906
216	FT	0.1557	345	0.1681	182	MS	0.2197	487	0.2373
217	DY	0.1552	344	0.1676	268	MT	0.1101	244	0.1189
218	EH	0.1552	344	0.1676	293	MV	0.0884	196	–
219	IR	0.1543	342	0.1666	398	MW	0.0081	18	–
220	EY	0.1539	341	0.1661	384	MY	0.0217	48	–

Continued on next page

Table S2 (continued)

Sorted by relative frequency (rank)					Sorted alphabetically by dipeptide name				
Rank	Dipep-tide	Rel. freq. in DisProt [%]	Abs. occurr. in DisProt	Rel. freq. in dataset [%]	Rank	Dipep-tide	Rel. freq. in DisProt [%]	Abs. occurr. in DisProt	Rel. freq. in dataset [%]
221	QI	0.1530	339	0.1652	147	NA	0.2707	600	0.2923
222	HP	0.1521	337	0.1642	362	NC	0.0343	76	–
223	EM	0.1502	333	0.1622	167	ND	0.2346	520	0.2533
224	HL	0.1498	332	0.1618	136	NE	0.2978	660	0.3216
225	IN	0.1489	330	–	260	NF	0.1155	256	–
226	MD	0.1489	330	0.1608	125	NG	0.3276	726	0.3537
227	MG	0.1466	325	0.1583	304	NH	0.0772	171	–
228	FK	0.1462	324	0.1579	205	NI	0.1760	390	0.1900
229	FD	0.1462	324	0.1579	139	NK	0.2861	634	0.3089
230	GM	0.1457	323	0.1574	141	NL	0.2797	620	0.3021
231	KF	0.1417	314	0.1530	295	NM	0.0848	188	–
232	YE	0.1412	313	0.1525	143	NN	0.2784	617	0.3006
233	MK	0.1408	312	0.1520	145	NP	0.2743	608	0.2962
234	II	0.1403	311	–	173	NQ	0.2256	500	0.2436
235	FA	0.1394	309	0.1505	192	NR	0.1999	443	0.2158
236	AY	0.1376	305	0.1486	90	NS	0.4133	916	0.4463
237	HE	0.1372	304	0.1481	164	NT	0.2432	539	0.2626
238	PH	0.1367	303	0.1476	179	NV	0.2220	492	0.2397
239	SM	0.1340	297	0.1447	375	NW	0.0275	61	–
240	YD	0.1336	296	0.1442	285	NY	0.0952	211	–
241	AH	0.1331	295	0.1437	12	PA	0.7508	1664	0.8107
242	YP	0.1331	295	0.1437	318	PC	0.0605	134	0.0653
243	KH	0.1317	292	0.1423	133	PD	0.3041	674	0.3284
244	TF	0.1317	292	0.1423	30	PE	0.5865	1300	0.6334
245	PY	0.1308	290	0.1413	208	PF	0.1696	376	0.1832
246	MP	0.1299	288	0.1403	27	PG	0.6087	1349	0.6572
247	YL	0.1290	286	0.1393	238	PH	0.1367	303	0.1476
248	AM	0.1263	280	0.1364	195	PI	0.1940	430	0.2095
249	FQ	0.1236	274	0.1335	59	PK	0.4724	1047	0.5101
250	LH	0.1232	273	0.1330	67	PL	0.4503	998	0.4862
251	RH	0.1227	272	0.1325	257	PM	0.1182	262	0.1276
252	DH	0.1223	271	0.1320	174	PN	0.2251	499	0.2431
253	HA	0.1223	271	0.1320	7	PP	0.8464	1876	0.9140
254	VF	0.1209	268	0.1306	87	PQ	0.4210	933	0.4546
255	HD	0.1205	267	0.1301	105	PR	0.3713	823	0.4010
256	HK	0.1200	266	0.1296	8	PS	0.7995	1772	0.8633
257	PM	0.1182	262	0.1276	47	PT	0.5076	1125	0.5481
258	ML	0.1178	261	–	71	PV	0.4467	990	0.4823
259	KY	0.1169	259	0.1262	339	PW	0.0492	109	0.0531
260	NF	0.1155	256	–	245	PY	0.1308	290	0.1413
261	YN	0.1151	255	–	98	QA	0.3912	867	0.4224
262	QH	0.1142	253	0.1233	366	QC	0.0316	70	–
263	LY	0.1142	253	0.1233	165	QD	0.2369	525	0.2558
264	HR	0.1132	251	0.1223	79	QE	0.4340	962	0.4687
265	HQ	0.1132	251	0.1223	286	QF	0.0943	209	0.1018
266	KM	0.1128	250	0.1218	111	QG	0.3582	794	0.3868
267	YT	0.1119	248	–	262	QH	0.1142	253	0.1233
268	MT	0.1101	244	0.1189	221	QI	0.1530	339	0.1652
269	FV	0.1101	244	–	115	QK	0.3492	774	0.3771

Continued on next page

Table S2 (continued)

Sorted by relative frequency (rank)					Sorted alphabetically by dipeptide name				
Rank	Dipep-tide	Rel. freq. in DisProt [%]	Abs. occurr. in DisProt	Rel. freq. in dataset [%]	Rank	Dipep-tide	Rel. freq. in DisProt [%]	Abs. occurr. in DisProt	Rel. freq. in dataset [%]
270	HH	0.1092	242	0.1179	120	QL	0.3366	746	0.3635
271	FR	0.1087	241	0.1174	300	QM	0.0821	182	–
272	TY	0.1065	236	0.1150	183	QN	0.2193	486	0.2368
273	FN	0.1056	234	–	77	QP	0.4345	963	0.4692
274	HT	0.1051	233	0.1135	53	QQ	0.4846	1074	0.5233
275	YR	0.1047	232	0.1130	151	QR	0.2648	587	0.2860
276	TH	0.1042	231	0.1125	96	QS	0.3961	878	0.4278
277	DM	0.1024	227	–	152	QT	0.2617	580	0.2826
278	RF	0.1020	226	–	187	QV	0.2080	461	0.2246
279	LM	0.1011	224	–	368	QW	0.0307	68	–
280	YQ	0.0997	221	0.1077	291	QY	0.0889	197	–
281	YK	0.0993	220	0.1072	114	RA	0.3546	786	0.3829
282	CS	0.0993	220	–	354	RC	0.0433	96	–
283	YA	0.0988	219	–	150	RD	0.2653	588	0.2865
284	VH	0.0970	215	0.1047	97	RE	0.3948	875	0.4263
285	NY	0.0952	211	–	278	RF	0.1020	226	–
286	QF	0.0943	209	0.1018	82	RG	0.4277	948	0.4619
287	TM	0.0938	208	0.1013	251	RH	0.1227	272	0.1325
288	MN	0.0925	205	–	201	RI	0.1850	410	0.1998
289	HV	0.0902	200	–	83	RK	0.4264	945	0.4604
290	YV	0.0889	197	–	122	RL	0.3307	733	0.3571
291	QY	0.0889	197	–	296	RM	0.0839	186	–
292	SC	0.0884	196	–	181	RN	0.2202	488	0.2378
293	MV	0.0884	196	–	107	RP	0.3673	814	0.3966
294	RY	0.0880	195	–	148	RQ	0.2685	595	0.2899
295	NM	0.0848	188	–	44	RR	0.5175	1147	0.5588
296	RM	0.0839	186	–	50	RS	0.4986	1105	0.5384
297	MR	0.0839	186	0.0906	137	RT	0.2924	648	0.3157
298	HN	0.0835	185	–	184	RV	0.2166	480	0.2339
299	MQ	0.0830	184	–	356	RW	0.0370	82	–
300	QM	0.0821	182	–	294	RY	0.0880	195	–
301	VY	0.0812	180	–	14	SA	0.7178	1591	0.7751
302	FI	0.0794	176	–	292	SC	0.0884	196	–
303	FF	0.0790	175	–	31	SD	0.5843	1295	0.6309
304	NH	0.0772	171	–	17	SE	0.6786	1504	0.7328
305	IF	0.0753	167	–	159	SF	0.2486	551	0.2684
306	CL	0.0735	163	–	9	SG	0.7927	1757	0.8560
307	VM	0.0708	157	–	196	SH	0.1936	429	0.2090
308	IH	0.0690	153	–	149	SI	0.2680	594	0.2894
309	CG	0.0663	147	–	36	SK	0.5708	1265	0.6163
310	CP	0.0659	146	0.0711	28	SL	0.6055	1342	0.6538
311	LC	0.0654	145	–	239	SM	0.1340	297	0.1447
312	YI	0.0654	145	–	110	SN	0.3659	811	0.3951
313	YY	0.0641	142	–	5	SP	0.8816	1954	0.9520
314	MI	0.0641	142	–	68	SQ	0.4489	995	0.4848
315	GC	0.0627	139	–	40	SR	0.5369	1190	0.5798
316	IY	0.0623	138	–	2	SS	1.3224	2931	1.4280
317	IM	0.0618	137	–	32	ST	0.5811	1288	0.6275
318	PC	0.0605	134	0.0653	84	SV	0.4232	938	0.4570

Continued on next page

Table S2 (continued)

Sorted by relative frequency (rank)					Sorted alphabetically by dipeptide name				
Rank	Dipep-tide	Rel. freq. in DisProt [%]	Abs. occurr. in DisProt	Rel. freq. in dataset [%]	Rank	Dipep-tide	Rel. freq. in DisProt [%]	Abs. occurr. in DisProt	Rel. freq. in dataset [%]
319	WG	0.0600	133	–	326	SW	0.0528	117	–
320	HI	0.0596	132	–	180	SY	0.2206	489	0.2382
321	AW	0.0591	131	–	49	TA	0.5004	1109	0.5403
322	EC	0.0591	131	–	335	TC	0.0510	113	–
323	AC	0.0559	124	–	123	TD	0.3298	731	0.3561
324	HF	0.0550	122	–	60	TE	0.4715	1045	0.5091
325	YF	0.0541	120	–	244	TF	0.1317	292	0.1423
326	SW	0.0528	117	–	80	TG	0.4327	959	0.4672
327	FH	0.0528	117	–	276	TH	0.1042	231	0.1125
328	CT	0.0523	116	–	200	TI	0.1854	411	0.2002
329	GW	0.0523	116	–	100	TK	0.3853	854	0.4161
330	WE	0.0519	115	–	116	TL	0.3483	772	0.3761
331	CD	0.0514	114	–	287	TM	0.0938	208	0.1013
332	VC	0.0510	113	–	172	TN	0.2288	507	0.2470
333	CK	0.0510	113	–	38	TP	0.5473	1213	0.5910
334	FY	0.0510	113	–	156	TQ	0.2531	561	0.2733
335	TC	0.0510	113	–	160	TR	0.2482	550	0.2680
336	MF	0.0505	112	–	19	TS	0.6687	1482	0.7220
337	YH	0.0496	110	–	73	TT	0.4408	977	0.4760
338	WD	0.0496	110	–	129	TV	0.3154	699	0.3406
339	PW	0.0492	109	0.0531	365	TW	0.0325	72	–
340	WA	0.0492	109	–	272	TY	0.1065	236	0.1150
341	DW	0.0487	108	–	92	VA	0.4115	912	0.4443
342	KC	0.0487	108	–	332	VC	0.0510	113	–
343	MM	0.0483	107	–	130	VD	0.3136	695	0.3386
344	CE	0.0474	105	–	78	VE	0.4340	962	0.4687
345	WS	0.0469	104	–	254	VF	0.1209	268	0.1306
346	CR	0.0465	103	–	126	VG	0.3249	720	0.3508
347	EW	0.0465	103	–	284	VH	0.0970	215	0.1047
348	WL	0.0456	101	–	210	VI	0.1687	374	0.1822
349	DC	0.0456	101	–	121	VK	0.3352	743	0.3620
350	CA	0.0456	101	–	132	VL	0.3046	675	0.3289
351	CV	0.0442	98	–	307	VM	0.0708	157	–
352	HY	0.0438	97	–	178	VN	0.2224	493	0.2402
353	WK	0.0433	96	–	70	VP	0.4467	990	0.4823
354	RC	0.0433	96	–	161	VQ	0.2463	546	0.2660
355	HM	0.0379	84	–	186	VR	0.2089	463	0.2256
356	RW	0.0370	82	–	65	VS	0.4521	1002	0.4882
357	WR	0.0370	82	–	118	VT	0.3438	762	0.3712
358	LW	0.0365	81	–	131	VV	0.3050	676	0.3293
359	WN	0.0365	81	–	371	VW	0.0302	67	–
360	MH	0.0361	80	–	301	VY	0.0812	180	–
361	FM	0.0356	79	–	340	WA	0.0492	109	–
362	NC	0.0343	76	–	399	WC	0.0072	16	–
363	CQ	0.0334	74	–	338	WD	0.0496	110	–
364	WT	0.0325	72	–	330	WE	0.0519	115	–
365	TW	0.0325	72	–	386	WF	0.0208	46	–
366	QC	0.0316	70	–	319	WG	0.0600	133	–
367	WQ	0.0316	70	–	393	WH	0.0131	29	–

Continued on next page

Table S2 (continued)

Sorted by relative frequency (rank)					Sorted alphabetically by dipeptide name				
Rank	Dipep-tide	Rel. freq. in DisProt [%]	Abs. occurr. in DisProt	Rel. freq. in dataset [%]	Rank	Dipep-tide	Rel. freq. in DisProt [%]	Abs. occurr. in DisProt	Rel. freq. in dataset [%]
368	QW	0.0307	68	–	389	WI	0.0167	37	–
369	CN	0.0307	68	–	353	WK	0.0433	96	–
370	YM	0.0302	67	–	348	WL	0.0456	101	–
371	VW	0.0302	67	–	395	WM	0.0104	23	–
372	CI	0.0298	66	–	359	WN	0.0365	81	–
373	KW	0.0298	66	–	377	WP	0.0257	57	0.0278
374	IC	0.0289	64	–	367	WQ	0.0316	70	–
375	NW	0.0275	61	–	357	WR	0.0370	82	–
376	FC	0.0266	59	–	345	WS	0.0469	104	–
377	WP	0.0257	57	0.0278	364	WT	0.0325	72	–
378	YC	0.0257	57	–	380	WV	0.0248	55	–
379	IW	0.0248	55	–	396	WW	0.0099	22	–
380	WV	0.0248	55	–	392	WY	0.0135	30	–
381	HC	0.0244	54	–	283	YA	0.0988	219	–
382	CF	0.0235	52	–	378	YC	0.0257	57	–
383	CC	0.0230	51	–	240	YD	0.1336	296	0.1442
384	MY	0.0217	48	–	232	YE	0.1412	313	0.1525
385	CY	0.0217	48	–	325	YF	0.0541	120	–
386	WF	0.0208	46	–	193	YG	0.1990	441	0.2149
387	CH	0.0180	40	–	337	YH	0.0496	110	–
388	HW	0.0171	38	–	312	YI	0.0654	145	–
389	WI	0.0167	37	–	281	YK	0.0993	220	0.1072
390	CM	0.0162	36	–	247	YL	0.1290	286	0.1393
391	FW	0.0149	33	–	370	YM	0.0302	67	–
392	WY	0.0135	30	–	261	YN	0.1151	255	–
393	WH	0.0131	29	–	242	YP	0.1331	295	0.1437
394	MC	0.0113	25	–	280	YQ	0.0997	221	0.1077
395	WM	0.0104	23	–	275	YR	0.1047	232	0.1130
396	WW	0.0099	22	–	170	YS	0.2310	512	0.2494
397	YW	0.0099	22	–	267	YT	0.1119	248	–
398	MW	0.0081	18	–	290	YV	0.0889	197	–
399	WC	0.0072	16	–	397	YW	0.0099	22	–
400	CW	0.0054	12	–	313	YY	0.0641	142	–

Table S3. Average chemical shift differences between protonated (pH ≈ 3) and deprotonated (pH ≈ 6) ASP and GLU residues. The protonation-dependent absolute differences were calculated separately as $\Delta\delta = |\delta_{prot} - \delta_{deprot}|$ for ^{1}H, ^{13}C, and ^{15}N, and averaged over all corresponding nuclei (n) in dipeptides containing ASP or GLU. All reported differences exceed the typical assignment uncertainties of the respective nuclei (^{1}H: 0.01 ppm, ^{13}C: 0.05 ppm, and ^{15}N: 0.1 ppm).

	^{1}H		^{13}C		^{15}N	
**Average over**	$\Delta\delta$ [ppm]	**n**	$\Delta\delta$ [ppm]	**n**	$\Delta\delta$ [ppm]	**n**
ASP	0.2544 ± 0.1224	140	2.7418 ± 1.0126	140	3.5798 ± 2.3160	35
GLU	0.1128 ± 0.0669	216	2.0175 ± 1.3897	180	0.6842 ± 0.2202	36
ASP and GLU	0.1685 ± 0.1157	356	2.3344 ± 1.2900	320	2.1116 ± 2.1827	71

Table S4. Average chemical shift differences determined for the partner (non-GLU/ASP) residues in dipeptides containing ASP or GLU. Differences measured between dipeptides with protonated (pH ≈ 3) and deprotonated (pH ≈ 6) ASP or GLU side-chain carboxyl groups. Absolute differences were calculated for ^{1}H, ^{13}C, and ^{15}N as $\Delta\delta = |\delta_{prot} - \delta_{deprot}|$ and averaged over all corresponding nuclei (n). All reported differences exceed the typical assignment uncertainties of the respective nuclei (^{1}H: 0.01 ppm, ^{13}C: 0.05 ppm, and ^{15}N: 0.1 ppm).

	^{1}H		^{13}C		^{15}N	
**Average over**	$\Delta\delta$ [ppm]	**n**	$\Delta\delta$ [ppm]	**n**	$\Delta\delta$ [ppm]	**n**
ASP	0.0340 ± 0.0459	292	0.2335 ± 0.2979	225	0.2228 ± 0.1789	66
GLU	0.0138 ± 0.0213	302	0.0780 ± 0.1489	232	0.1278 ± 0.0972	66
ASP and GLU	0.0237 ± 0.0370	594	0.1546 ± 0.2470	457	0.1753 ± 0.1516	132

References

1. Aspromonte, M. C. *et al.* DisProt in 2024: Improving function annotation of intrinsically disordered proteins. *Nucleic Acids Res.* **52**, D434–D441 (2024).

2. Nugnes, M. V. *et al.* DisProt in 2026: enhancing intrinsically disordered proteins accessibility, deposition, and annotation. *Nucleic Acids Res.* **54**, D383–D392 (2026).

3. Hoch, J. C. *et al.* Biological magnetic resonance data bank. *Nucleic Acids Res.* **51**, D368–D376 (2023).
